# *gone early*, a Novel Germline Factor, Ensures the Proper Size of the Stem Cell Precursor Pool in the *Drosophila* Ovary

**DOI:** 10.1371/journal.pone.0113423

**Published:** 2014-11-24

**Authors:** Shinya Matsuoka, Swati Gupta, Emiko Suzuki, Yasushi Hiromi, Miho Asaoka

**Affiliations:** 1 Department of Developmental Genetics, National Institute of Genetics, Mishima, Shizuoka, Japan; 2 Department of Genetics, SOKENDAI, Mishima, Shizuoka, Japan; 3 Structural Biology Center, National Institute of Genetics, Mishima, Shizuoka, Japan; University of Bern, Switzerland

## Abstract

In order to sustain lifelong production of gametes, many animals have evolved a stem cell–based gametogenic program. In the *Drosophila* ovary, germline stem cells (GSCs) arise from a pool of primordial germ cells (PGCs) that remain undifferentiated even after gametogenesis has initiated. The decision of PGCs to differentiate or remain undifferentiated is regulated by somatic stromal cells: specifically, epidermal growth factor receptor (EGFR) signaling activated in the stromal cells determines the fraction of germ cells that remain undifferentiated by shaping a Decapentaplegic (Dpp) gradient that represses PGC differentiation. However, little is known about the contribution of germ cells to this process. Here we show that a novel germline factor, Gone early (Goe), limits the fraction of PGCs that initiate gametogenesis. *goe* encodes a non-peptidase homologue of the Neprilysin family metalloendopeptidases. At the onset of gametogenesis, Goe was localized on the germ cell membrane in the ovary, suggesting that it functions in a peptidase-independent manner in cell–cell communication at the cell surface. Overexpression of Goe in the germline decreased the number of PGCs that enter the gametogenic pathway, thereby increasing the proportion of undifferentiated PGCs. Inversely, depletion of Goe increased the number of PGCs initiating differentiation. Excess PGC differentiation in the *goe* mutant was augmented by halving the dose of *argos*, a somatically expressed inhibitor of EGFR signaling. This increase in PGC differentiation resulted in a massive decrease in the number of undifferentiated PGCs, and ultimately led to insufficient formation of GSCs. Thus, acting cooperatively with a somatic regulator of EGFR signaling, the germline factor *goe* plays a critical role in securing the proper size of the GSC precursor pool. Because *goe* can suppress EGFR signaling activity and is expressed in EGF-producing cells in various tissues, *goe* may function by attenuating EGFR signaling, and thereby affecting the stromal environment.

## Introduction

Animals have developed various strategies for continuously producing gametes. In *Drosophila* and mouse, this is achieved by implementing two developmental pathways: direct gamete production from undifferentiated primordial germ cells (PGCs), and lifelong production of gametes from germline stem cells (GSCs) [1], [2]. GSCs arise from a subset of PGCs; allocation of some PGCs to a special microenvironment, called the niche, establishes their identity as GSCs [3]. In the *Drosophila* ovary, the direct gametogenesis pathway is triggered before GSC establishment [1], [4]–[6]; therefore, a subset of PGCs must somehow resist the overtly differentiating environment and remain in an undifferentiated state as GSC precursors. However, we know little about how the size of the GSC precursor pool is regulated.

**Figure 1:**
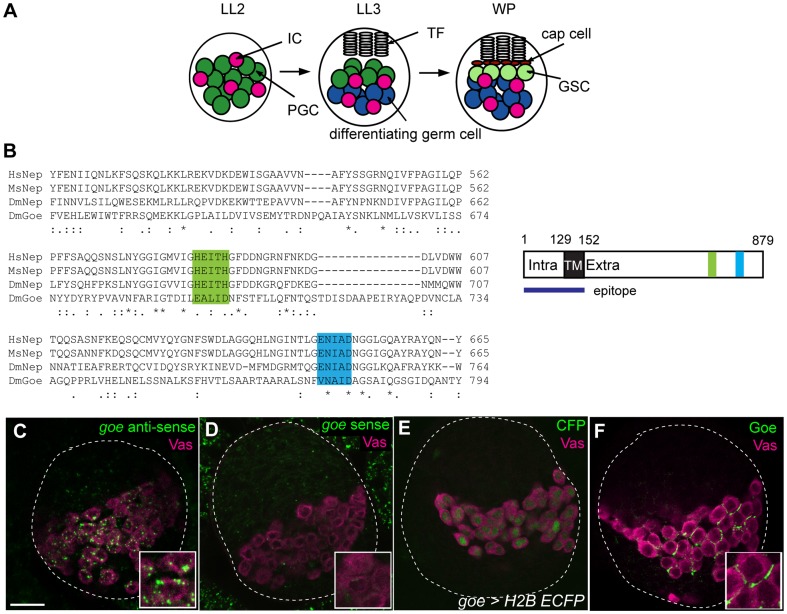
Gone early, a non-peptidase homologue of Neprilysin metalloendopeptidases, is expressed in germline cells of LL3 ovaries. (A) Key stages of PGC development. LL2, LL3, and WP are late second instar larval stage (72 h AEL [after egg laying]), late third instar larval stage (114 h AEL), and white pupal stage (120 h AEL), respectively. (B) Alignment of zinc-binding motifs of Gone early (Goe) with those of Neprilysin (Nep) from *Homo sapiens* (Hs), *Mus musculus* (Ms), and *Drosophila melanogaster* (Dm). The alignment was generated using the ClustalW algorithm. The symbols under the residues show Gonnet PAM 250 matrix scores: *, perfect match;: (colon),>0.5;. (period), ≤0.5. Amino-acid similarities between Goe and Hs, Ms, and Dm Nep are 38%, 37%, and 39%, respectively (between Hs Nep and Dm Nep: 59%). Two motifs are critical for the enzymatic activity of Neprilysin [34]: HExxH (green), which contains two His residues that serve as zinc ligands and a Glu residue functioning in catalysis; and ExxA/GD (blue), which contains a Glu that serves as the third zinc ligand. Gone early lacks both of these motifs. (C–F) All confocal images depict LL3 ovaries. (C) An ovary stained for *gone early* (*goe*) mRNA and the germline marker Vasa (Vas, magenta). *goe* mRNA was detected in the germline. (D) Sense probe control. Insets in C and D show magnified views of germ cells. (E) An ovary stained for CFP (green, *goe>H2B-ECFP*) and Vasa (magenta). CFP expression driven by *goe-gal4* (*goe>H2B-ECFP*) was found in the germline alone. For the enhancer element of *goe-gal4*, see Figure 3A. (F) An ovary stained with antibody against the intracellular and transmembrane domains of Goe (green) and anti-Vasa antibody (magenta). Anti-Vasa stained the cytoplasm of germ cells, whereas anti-Goe stained outside of germ cell cytoplasm, indicating that Goe was localized to the plasma membrane of germ cells. Goe was not distributed evenly on the germ cell plasma membrane, but was localized to sub-compartments of the membrane where neighboring germ cells face each other. The epitope used for antibody generation is shown in Figure 1B. White dashed lines in C–F outline whole ovaries. Anterior is up. Scale bar: 20 µm.

The timing and location of gametogenesis is controlled by the somatic environment of the PGCs. Somatic stromal cells called intermingled cells (ICs) contact PGCs in the center of the larval ovary, called the germ cell/IC (GC/IC) region, and maintain PGCs in an undifferentiated, proliferating state (Figure 1A) [6]. In the mid–third larval instar stage, a temporal signal delivered by the steroid hormone ecdysone activates a signaling pathway in the somatic cells that triggers niche formation and initiation of GSC establishment, as well as the induction of PGC differentiation via the direct gametogenesis pathway, in the late third larval instar stage (LL3) [5]. The somatic environment also controls spatial aspects of direct gametogenesis. PGC differentiation does not initiate uniformly throughout LL3 ovaries; rather, differentiating PGCs are located mostly in the posterior part of the GC/IC region, whereas PGCs in the anterior region remain undifferentiated (Figure 1A) [4]. This difference in PGC behavior along the anterior–posterior axis of the ovary likely results from a locally produced diffusible morphogen, Decapentaplegic (Dpp, a BMP2/4 homologue). This factor is produced by the anterior somatic cells [7], and is received by the anteriorly located PGCs, protecting them from gametogenesis by repressing the transcription of a differentiation gene, *bag of marbles* (*bam*). By contrast, posterior PGCs fail to receive Dpp, and therefore permit *bam* expression and initiate differentiation [7]–[10]. At the white pupal stage (WP), when GSC niche formation is complete (as evidenced by the appearance of cap cells), some of the anterior PGCs are accommodated in this niche and start asymmetric division as GSCs (Figure 1A) [1], [4], [6]. Thus, it is the shape of the Dpp signaling gradient that determines the size of the GSC precursor pool by protecting PGCs from the global differentiation signal ecdysone. Artificially induced excess PGC differentiation at the onset of gametogenesis results in a decrease or absence of GSCs in the adult GSC niche, underscoring the importance of regulation of PGC pool size [7], [11].

Previous work showed that EGFR signaling, activated in ICs, regulates the shape of the Dpp signaling gradient in the GC/IC region, thereby defining the fraction of PGCs that initiate differentiation [7]. The level of EGFR signaling, which is activated evenly among ICs, defines the number of ICs expressing the cell-surface proteoglycan Dally, which is required for Dpp movement from the signaling source; in addition, Dally stabilizes Dpp [7], [12], [13]. The number of ICs expressing Dally directly reflects the expansion of the Dpp signaling area [7]. Thus, the size of the PGC pool in LL3 ovaries is defined by the level of EGFR signaling in the somatic stromal environment. However, it remains unknown whether germ cells also actively participate in this process.

Here we show that germ cells express a novel integral membrane protein, Gone early (Goe), which contributes to the regulation of PGC pool size. Goe is expressed on the germ cell plasma membrane in LL3 ovaries. Overexpression and loss-of-function studies revealed that Goe prevents PGCs from entering the direct gametogenesis pathway. Because *goe* is expressed in various tissues and cells that produce EGF ligands, and its extracellular domain has the ability to attenuate EGFR signaling activity, Goe may act in the extracellular matrix to affect EGFR signaling in neighboring stromal ICs.

## Materials and Methods

### Fly strains

*y w* was used as a wild-type strain. The following mutants and transgenic lines were used: *nos-gal4VP16* [14] from R. Lehmann (New York University, New York, NY, USA); *nub-gal4* and *ap-gal4* [15] from T. Hayashi (NIG, Mishima, Japan); *UAS-H2B-ECFP* [16] from S. Kondo (NIG, Mishima, Japan); *UAS-EgfrDN* [17] from M. Freeman (MRC Laboratory of Molecular Biology, Cambridge, UK); *UAS-pav-GFP* [18] from Y. M. Yamashita (University of Michigan, Ann Arbor, MI, USA); *bam-GFP* [19] from D. McKearin (HHMI, Chevy Chase, MD, USA); and *argos^delta7^* [20] from the Bloomington Stock Center (Indiana University, Bloomington, IN, USA). *goe^5–11^* and *goe^331^* alleles were generated by imprecise excision of a P element, EY01697, inserted in the 5′UTR of *goe*.

### Generation of *gone early* transgenic flies

Transgenic flies harboring *UASt-goe*, *UASt-goe-FLAG, UASp-goe*, *UASp-goe-FLAG*, *UASt-goe Intra*, *UASt-goe Extra-FLAG*, and *goe-gal4* were generated. For *UASt-goe* and *UASt-goe-FLAG*, the *goe* cDNA fragment (453 bp of 5′UTR and the full-length *goe* coding region) with or without the stop codon was amplified from LD21405 (BDGP Gold cDNA Collection) using the following primer sets: 5′-TATGCGGCCGCCCGTTTAAAAATT-3′ (*goe*-NotI primer) and 5′-TATCTCGAGTGTGTGAAAAGTCATTC-3′ (*goe*-XhoI primer) or 5′-TATCTCGAGGAGCAGGTTGGAGCAAGTC-3′ (*goe*-XhoI-FLAG primer). The resultant amplicon was subcloned into the *Not*I/*Xho*I sites of vector pUASt [21] or pUASt-FLAG (a gift from K. Emoto, Osaka Bioscience Institute, Osaka, Japan). For *UASp-goe*, the *goe* cDNA insert from *UASt-goe* was subcloned into the *Xba*I/*Not*I sites of pUASp [22]. For *UASp-goe-FLAG*, the *goe* cDNA fragment with the FLAG epitope was amplified from *UASt-goe-FLAG* using *goe*-NotI and *goe*-XbaI-FLAG (5′CACAAAGATCCTCTAGATTACTTG-3′) primers, and subcloned into the *Not*I/*Xba*I sites of pUASp. For *UASt-goe Intra* and *UASt-goe Extra-FLAG*, *goe* cDNA fragments lacking the extracellular domain (corresponding to amino acids 152–879) or intracellular domain (corresponding to amino acids 1–117) were subcloned into pUASp using the In-Fusion Advantage PCR Cloning Kit (Clontech). Each insert was amplified using *goe*-NotI and *goe*-XhoI primers (for *UASt-goe Intra*) or *goe*-NotI and *goe*-XhoI-FLAG primers (for *UASt-goe Extra-FLAG*), and then subcloned into the *Not*I/*Xho*I sites of pUASt or pUASt-FLAG, respectively. For *goe-gal4*, a putative enhancer element (sequences from position −278 to position +6077 relative to transcription start site) was amplified from the genomic DNA of strain *y w* using the following primers: 5′-TATGCGGCCGCAAAATTAAAGAAGTGTGTGCC-3′ (*goe-gal4* forward primer) and 5′-TATGCGGCCGCTGCGTTTGGATGTGCAACTC-3′ (*goe-gal4* reverse primer). The *Not*I-*Not*I fragment of the resultant amplicon was subcloned into vector pWGAL4. All constructs were confirmed by sequencing before they were introduced into flies. Germline transformation was performed as described previously [23], using *y w* embryos as recipients.

### Staging

To minimize individual variation due to developmental or nutritional conditions, larvae were carefully cultured in under-crowded conditions: eggs from 50 flies were collected on normal food for 2 hours, and subsequently cultured at 25 degrees. To obtain LL2 ovaries, larvae at 72 h after egg-laying (AEL), which remained within the food, were dissected. To obtain LL3 ovaries, larvae at 114 h AEL, which were slowly wandering out of the food but had not yet initiated puparium formation, were dissected. At this stage, most terminal filaments were finished stacking. To obtain WP ovaries, white pupae at 120 h AEL, which had already started puparium formation with everted anterior spiracles but still had a soft white puparium, were dissected. At this stage, cap cells appeared at the base of each terminal filament.

### Anti-Goe antibody generation and immunohistochemistry

To generate the anti-Goe antibody, a DNA fragment encoding the intracellular and transmembrane regions of Goe protein (amino acid residues 1–150) was cloned into vector pET-21a(+) (Novagen) to produce His_6_-tagged protein. The protein was expressed in BL21 bacteria and purified with Ni-NTA resin (QIAGEN). Polyclonal antiserum was generated in rabbits, and then affinity-purified with antigen (MBL Co., Ltd). The specificity of Goe antiserum was verified by immunostaining and Western blotting of the *y w* control and *goe^331/331^* mutant.

Immunostaining of LL2, LL3, and WP ovaries was carried out as described previously [7]. Wing imaginal discs were dissected in PBS and fixed in fixation solution (50 mM EDTA, 8% formaldehyde in PBS) for 30 min at room temperature, and then washed and stained with the same method used for the ovary. The following primary antibodies were used: mouse anti-Hts 1B1 (1∶20), mouse anti-Engrailed 4D9 (1∶2) (Developmental Studies Hybridoma Bank at the University of Iowa), rabbit anti-Vasa (1∶1000) (a gift from Satoru Kobayashi, NIBB, Okazaki, Japan), rat anti-Vasa (1∶1000) (a gift from Akira Nakamura, RIKEN, Kobe, Japan), rabbit anti-GFP (1∶500) (A11122, Invitrogen), rat anti-GFP (1∶250) (D153-3, MBL), mouse anti-dpERK (1∶200) (M8159, Sigma), rabbit anti–phospho-Histone H3 (1∶500) (#06-570, Millipore), and rabbit anti-Goe (1∶1000, this study). FITC-, Cy3-, and Cy5-conjugated secondary antibodies were used at 1∶400 (Jackson ImmunoResearch). Stained samples were mounted in Vectashield (H-1200, Vector laboratories). For the TUNEL assay, apoptotic cells were detected using the ApopTag Plus In Situ Apoptosis Fluorescein Detection Kit (S7111, Millipore). Images were collected using a Zeiss LSM5 Pascal confocal microscope (Zeiss), as described previously [7]. Images were minimally and equally enhanced by adjusting brightness and contrast of whole images using Zeiss LSM 5 Image Browser or Adobe Photoshop CS.

### *In situ* hybridization

DIG-labeled probes were synthesized from cDNA plasmids obtained from the BDGP collection (*gone early*: LD21405; *argos*, RE21614) using DIG RNA labeling mix (#11277073910, Roche). Labeled probes were cut into 300-bp fragments by alkaline hydrolysis. Double staining of ovaries by *in situ* hybridization and immunostaining with anti-Vasa antibody was performed as described previously [7].

### Quantification of PGC differentiation and the number of GSCs

**Figure 2:**
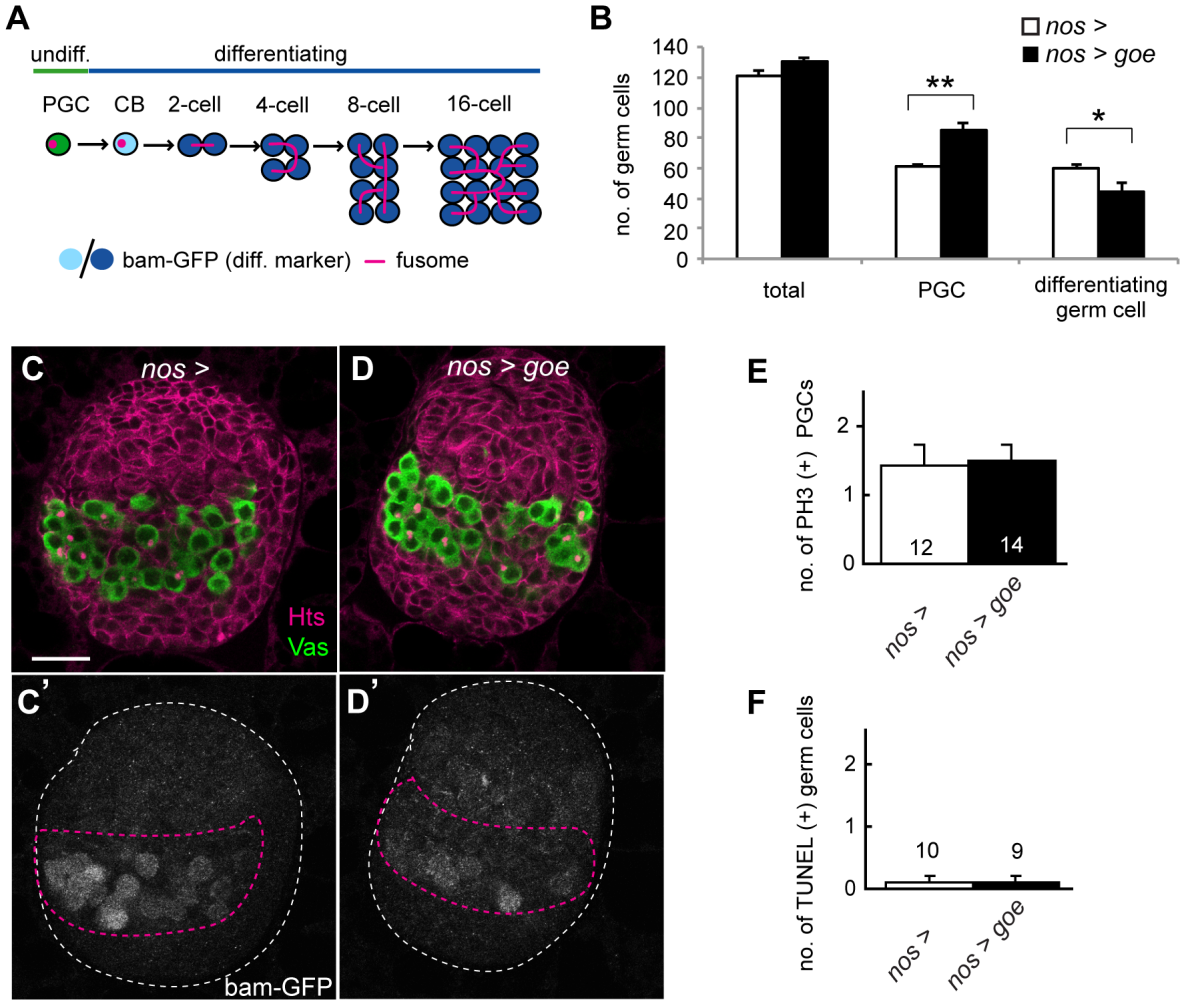
Over-expression of *gone early* attenuates PGC differentiation in LL3 ovaries. (A) A schematic view of PGC differentiation. Differentiating germ cells are distinguished from PGCs by *bam-GFP* expression (see Materials and Methods). Differentiating germ cells are further categorized into cystoblasts (CBs) and 2-cell, 4-cell, 8-cell, or 16-cell cysts depending on fusome morphology. (B) Average numbers of total germ cells, PGCs, and differentiating germ cells (CB to 16-cell cysts) in LL3 ovaries overexpressing *goe* in the germline (black bars, *nos>goe*) and control ovaries carrying *nos-gal4* alone (white bars, *nos*>). Ten ovaries are examined for each genotype. P values were calculated using the U-test (**p<0.0005, *p<0.03). No significant difference was observed in total germ cell numbers (p = 0.11, U-test). (C–D′) All confocal images depict LL3 ovaries triple-stained for Vasa, Hts, and GFP (*bam-GFP*). (C, D) Anti-Vasa labeled germ cells (green), and anti-Hts outlined somatic cells and fusomes (magenta). (C′, D′) Differentiating germ cells were marked by *bam-GFP*. (C, C′) A *nos*> control ovary. (D, D′) A *nos>goe* ovary. White and magenta dashed lines outline whole ovaries and GC/IC regions, respectively. Anterior is up. (E) Average numbers of phospho-histone H3 (PH3)-positive PGCs in LL3 ovaries. No significant difference was observed between the *nos>goe* ovaries and the *nos*>control ovaries (p = 0.82, U-test). The number of ovaries examined is shown at the bottom of each bar. (F) Average numbers of TUNEL-positive germ cells in LL3 ovaries. No significant difference in the number of apoptotic germ cells was observed between the *nos>goe* and the *nos*> control ovaries (p = 0.94, U-test). The number of ovaries examined is indicated over each bar. Error bars in E and F indicate SEM. Scale bar: 20 µm.

For PGC differentiation analyses in LL3 ovaries, differentiating germ cells were distinguished from PGCs by their expression of *bam-GFP*, as described before [7]. Briefly, to determine whether a cell expressed *bam-GFP*, we analyzed serial confocal sections (0.58 µm each, about 15 sections per germ cell) of ovaries triple-stained for GFP (*bam-GFP*), Hts, and Vasa using the Zeiss LSM 5 Image Browser and ImageJ. *bam-GFP*-positive cells were considered to be differentiating germ cells, whereas *bam-GFP*-negative cells were considered to be PGCs. Differentiating germ cells were further classified as cystoblasts (CBs) or 2-cell, 4-cell, 8-cell, or 16-cell cysts based on fusome morphology and intensity of *bam-GFP* expression [24] (Figure 2A). CB, 2-cell, 4-cell, and 8–16 cell cysts contain spherical, dumbbell-shaped, U-shaped, and branched fusomes, respectively. *bam-GFP* expression is low in CBs and 2-cell cysts, but more intense in 4–16 cell cysts [7], [19]. *bam-GFP*–negative germ cells contained either spherical or dumbbell-shaped fusomes, but never contained U-shaped or branched fusomes. *bam-GFP*–negative germ cells with spherical or dumbbell-shaped fusomes were classified as single or dividing PGCs, respectively. Each cyst was counted as one differentiating germ cell, and dividing PGCs were counted as two PGCs. The total number of germ cells was calculated as the sum of PGCs and differentiating germ cells.

For GSC analysis in WP ovaries, flies carrying the *bam-GFP* marker were stained with anti-Engrailed and anti-Vasa antibodies to label cap cells and germ cells, respectively. Ovarioles were classified according to the number of GSCs, defined as Vasa-positive, *bam-GFP*–negative cells contacting cap cells. In some ovarioles, only *bam-GFP*–positive cells were found in the niche, and these were scored as *bam-GFP*–positive ovarioles. P values were calculated using the chi-square test.

### Quantification of dedifferentiating germline cysts

When germline cysts undergo dedifferentiation, ring canals that connect each germ cell are completely closed, exhibiting a dot-like structure called the ring canal remnant [25]. The ring canal remnant is marked by its components, such as Anillin [25], [26] and Pav-GFP [27], [28]. A dot-like fusome accumulated in the ring canal remnant (Figure S2). Because a similar dot-like fusome is also detected in the proliferating PGCs [29], “dedifferentiating germline cysts” were defined as germline cysts containing a dot-like fusome that were also *bam-GFP* positive. P values were calculated using the U-test.

### Electron Microscopy

LL3 ovaries were dissected from larvae in EBR (130 mM NaCl, 5 mM KCl, 2 mM CaCl_2_, 10 mM HEPES [pH 6.9]), and then fixed for several hours at room temperature in 2% paraformaldehyde and 2.5% glutaraldehyde in 0.1 M sodium cacodylate buffer (pH 7.3). After the samples were rinsed three times for 5 min each in 0.1 M sodium cacodylate buffer containing 3% sucrose, they were fixed again for 1 h on ice in 1% OsO4 in 0.1 M sodium cacodylate buffer. After the samples were rinsed three times for 5 min each in ice-cold distilled water, they were stained *en bloc* with 0.5% aqueous uranyl acetate for 1 h on ice, and then dehydrated in an ethanol series (50%, 70%, 90% for 5 min each on ice, and then 99.5% three times for 5 min each at room temperature). The samples were then embedded in Epon 812 (TAAB) and cured at 70°C for 3 d. The embedded samples were cut into semi-thin sections (1 µm thick), stained with toluidine blue to select the areas of interest, and then cut into ultrathin sections (70–80 nm). These ultrathin sections were collected on copper grids and stained with 2% uranyl acetate and lead citrate [30]. Electron micrographs were obtained using a VELETA CCD camera (Olympus Soft Imaging Solutions) mounted on a JEM-1010 electron microscope (Jeol Ltd.)

## Results and Discussion

### Gone early, a non-peptidase homologue to Neprilysin family metalloendopeptidases, is expressed on germ cell membranes

Many organisms have a remarkable number of proteins with peptidase-like sequences that appear to lack the essential residues for catalytic activity [31]. These so-called non-peptidase homologues are assumed to play roles in regulating peptidase activity as inhibitors, or to mediate binding functions that no longer require peptidase activity; however, little is known about their roles in development [31]–[34]. One of the genes expressed in the *Drosophila* ovary at LL3, *CG9634* (hereafter referred to as *gone early* [*goe*]), is predicted to encode a novel non-peptidase homologue of the Neprilysin (M13) family peptidases [35]. Neprilysins are typically type II integral membrane glycoproteins with their active sites facing the extracellular environment [34]. Like other member of the Neprilysin family, *goe* encodes a type II transmembrane protein with short intracellular domain (129 residues) and a much longer extracellular domain, but lacks two consensus zinc-ion binding motifs, HExxH and ExxA/GD, that are essential for catalysis by the Neprilysin family peptidases (Figure 1B) [34].

**Figure 3:**
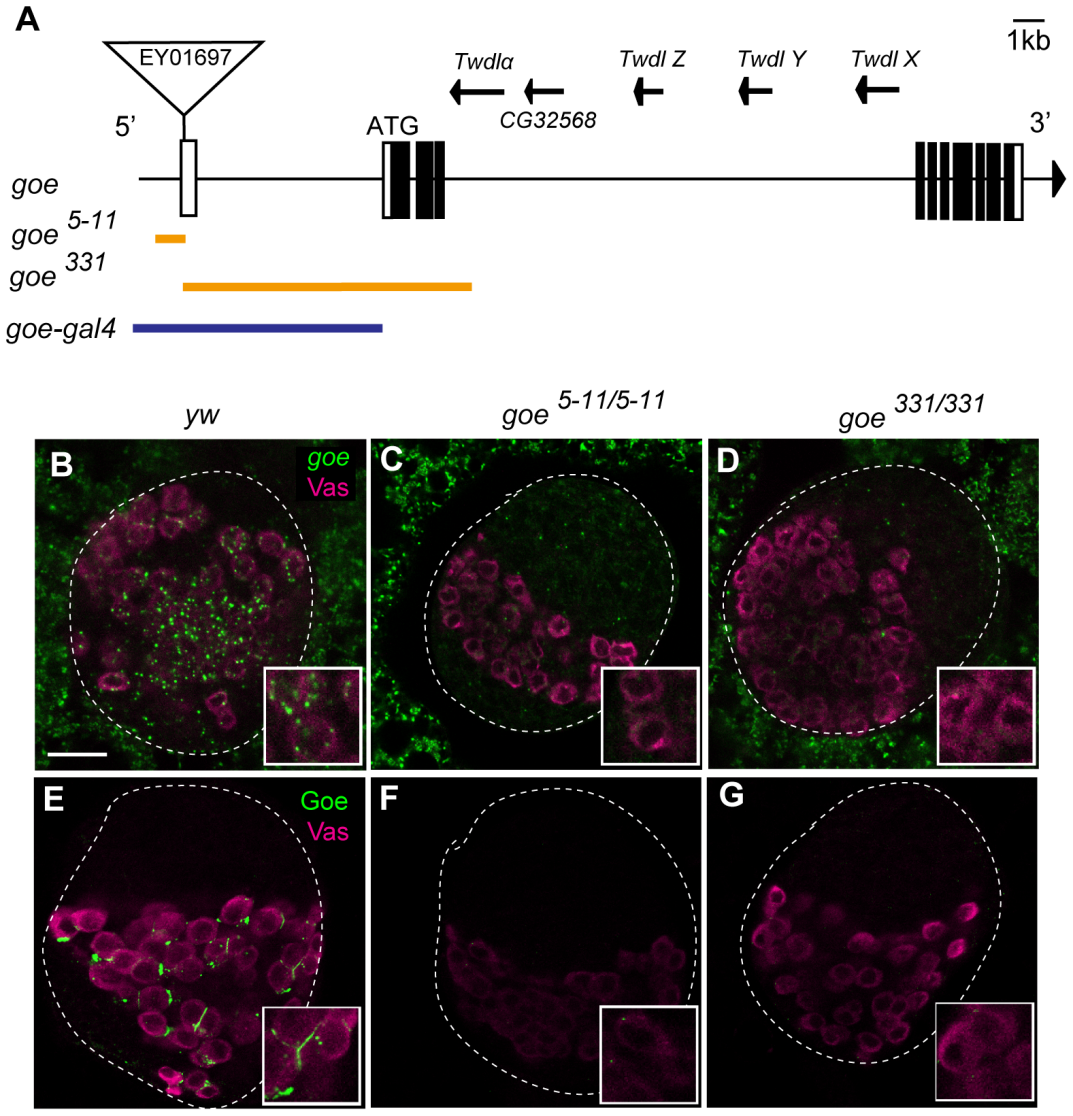
Generation of *gone early* mutants. (A) Schematic representation of the *goe* locus, consisting of *CG9634 (goe)*, and five previously annotated genes, *CG32568, Twdlalpha, TwdlX, TwdlY and TwdlZ*, which are located on the complimentary strand. White and black boxes represent the UTR and ORF of *goe*, respectively. Two *goe* alleles, *goe^5–11^* and *goe^331^*, were generated by imprecise excision of a P-element, EY01697, that was inserted in the first exon of *goe*. The detailed position of the P-element insertion is represented in CG9634 in FlyBase (http://flybase.org). Orange lines indicate deletions in *goe* mutants; *goe^5–11^* has a 92-bp deletion, which encompasses a 63-bp upstream genomic sequence and a part of exon 1, including the transcriptional start site. *goe^331^* has a 8229-bp deletion staring from 30 bp downstream of the transcription start site, including most of the 5′UTR and the translation start site. In this study, the transheterozygote *goe^5–11/331^* was used to preclude potential second-site mutations that might have been introduced during the excision event. The blue line indicates a 6355 bp genomic fragment used to make *goe-gal4* (see Materials and Methods). (B–G) All confocal images depict LL3 ovaries. (B, E) *y w* control. (C, F) *goe^5–11/5–11^*. (D, G) *goe^331/331^*. (B–D) Ovaries were stained for *goe* mRNA (green) and Vasa (magenta). No detectable *goe* mRNA was found in either *goe^5–11/5–11^* or *goe^331/331^* ovaries. (E–G) Ovaries were stained for Goe protein (green) and Vasa (magenta). No detectable Goe protein was seen in either *goe^5–11/5–11^* or *goe^331/331^* ovaries. Insets show magnified views of germ cells. White dashed lines outline whole ovaries. Anterior is up. Scale bar: 20 µm.

*goe* was expressed in a germline-specific manner in LL3 ovaries: *goe* mRNA was detected in germ cells, but not in somatic ICs (Figure 1C). Consistent with this, GFP expression under the control of the *goe-gal4*, a gal4 line controlled by the upstream genomic region of *goe* (Figure 3A), was also detected exclusively in germ cells (Figure 1E). Antibody against intracellular and transmembrane domains of Goe revealed that the Goe protein was not uniformly present on the plasma membranes of germ cells, but was instead localized to a membrane compartment at the interface between germ cells (Figure 1F). Such interfaces were often penetrated by thin cell processes of ICs (Figure S1), raising the possibility that Goe may act in a peptidase-independent manner to mediate communication between germ cells and ICs.

### *gone early* prevents excessive PGC differentiation in LL3 ovaries

To investigate the role of Goe on the germ cell plasma membrane in LL3 ovaries, we first examined the effects of overexpression of *goe* in the germline, using the Gal4/UAS system. When *goe* was over-expressed in germ cells using the germline-specific Gal4 driver *nos-gal4-VP16* [14] (*nos>goe*), we observed a decrease in the number of germ cells initiating gametogenesis. In contrast to control ovaries (*nos*>), these ovaries contained fewer cystoblasts and developing germline cysts (collectively called differentiating germ cells) (Figure 2B–D′, Figure S2), which can be distinguished from PGCs by the expression of a differentiation marker, *bam-GFP* [7], [8] (Figure 2A). Because the total number of germ cells was indistinguishable from control ovaries (U-test, P = 0.11) (Figure 2B), the decrease in the number of differentiating germ cells likely resulted from a reduction in the fraction of germ cells that were allocated to gametogenesis. In support of this, we detected a significant increase in the number of undifferentiated PGCs in *nos>goe* ovaries (Figure 2B). By contrast, the numbers of mitotic and apoptotic cells were essentially the same as those in control ovaries (Figure 2E, F), suggesting that overexpression of *goe* does not affect either proliferation or survival of germ cells. These results indicate that *goe* can suppress PGC differentiation in LL3 ovaries.

**Figure 4:**
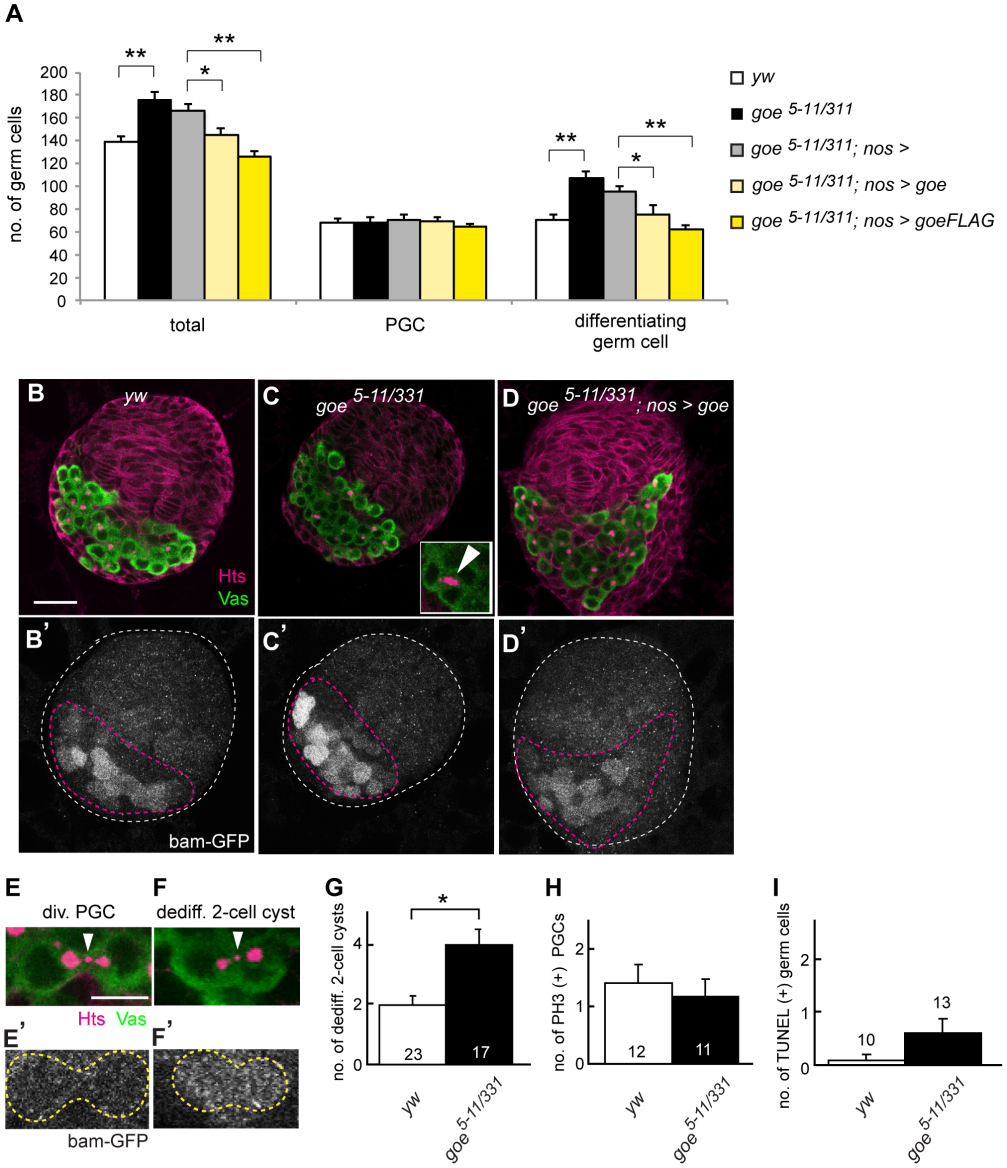
Absence of *gone early* causes an excessive PGC differentiation in LL3 ovaries. (A) Average numbers of total germ cells, PGCs, and differentiating germ cells in LL3 wild type (white bars, *y w*), *goe^5–11/331^* (black bars), *goe^5–11/331^* with *nos-gal4* alone (gray bars, *goe^5–11/331^*; *nos*>), *goe^5–11/331^* carrying *nos-gal4* and *UAS-goe* (pale yellow bars, *goe^5–11/331^*; *nos>goe*), or *UAS-goeFLAG* (yellow bars, *goe^5–11/331^*; *nos>goeFLAG*). Excessive PGC differentiation in the *goe* mutant was rescued when *goe* was expressed in germline by *nos-gal4* (compare *goe^5–11/331^; nos*> control with *goe^5–11/331^; nos>goe* and *goe^5–11/331^; nos>goe-FLAG*), indicating that the phenotype is due to loss of *goe* function. No significant difference in the number of PGCs was observed between these ovaries (p>0.2, U-test). Between 9 and 23 ovaries were examined for each data point. (**p<0.001, *p<0.04; U-test). (B–D′) All confocal images depict LL3 ovaries triple-stained for Vasa (green, B–D), Hts (magenta, B–D), and GFP (*bam-GFP*) (white, B′–D′). (B, B′) A *y w* control ovary. (C, C′) A *goe^5–11/331^* ovary. (D, D′) A rescued ovary (*goe^5–11/331^; nos>goe*). Inset in C shows a 4-cell cyst harboring a U-shaped fusome (white arrowhead). White and magenta dashed lines in B′–D′ outline whole ovaries and GC/IC regions, respectively. Anterior is up. (E–F′) Dedifferentiation of germline cysts can be identified by the presence of a dot-like fusome in the ring canal remnant and *bam-GFP* expression (see Materials and Methods and Figure S4) [25]. Comparison of a dividing PGC (E, E′) and a dedifferentiating 2-cell cyst (F, F′) at LL3. Anti-Vasa (green) and anti-Hts (magenta) mark the germ cell cytoplasm and fusome, respectively. Both the PGC undergoing cytokinesis (E) and the 2-cell cyst undergoing dedifferentiation (F) had a dot-like fusome (white arrowheads) in the ring canal remnants (Figure S2), but only the 2-cell cyst expressed *bam-GFP* (E′, F′). (G) Average numbers of dedifferentiating 2-cell cysts in LL3 ovaries. There were 2-fold more dedifferentiating 2-cell cysts in *goe^5–11/331^* ovaries than in *y w* control ovaries (*p = 0.002, U-test). At LL3, dedifferentiation was observed mainly in 2-cell cysts, as observed in adult testis [49]. (H) The average number of PH3-positive PGCs in LL3 ovaries. No significant difference in the number of dividing PGCs was observed between *goe^5–11/331^* and *y w* control ovaries (p = 0.58, U-test). (I) The average number of TUNEL-positive germ cells in LL3 ovaries. No significant difference in the number of apoptotic germ cells was found between *goe^5–11/331^* and *y w* control ovaries (p = 0.18, U-test). The total number of ovaries examined is indicated at the bottom of each bar (G, H) or over each bar (I). Error bars in G–I indicate SEM. Scale bar: 20 µm.

To determine whether *goe* is required to limit PGC differentiation at LL3, we next examined the effects of *goe* loss of function. To this end, we generated two deletion alleles, *goe^5–11^* and *goe^331^* (Figure 3A). In both cases, *goe* mRNA and Goe protein were undetectable (Figure 3B–G), indicating that these alleles are null or strong hypomorphs. Depletion of Goe resulted in expansion of the differentiating germ cell fraction (Figure 4A–C′, Figure S3A–B″). In *goe^5–11/331^* ovaries at LL3, we detected a large increase in the number of differentiating germ cells expressing *bam-GFP*, about 1.5-fold greater than the number in *y w* controls (Figure 4A). Because this phenotype could be completely rescued by inducing *goe* expression in the germline alone (compare *goe^5–11/331^; nos>goe* or *goe^5–11/331^; nos>goe-FLAG* with *y w* in Figure 4A, U-test, P = 0.44 and 0.13, respectively) (Figure 4D, D′, Figure S3C–C″), we concluded that Goe in the germline is necessary and sufficient to restrict the number of differentiating germ cells in LL3 ovaries.

If the loss of Goe simply shifts the PGC pool towards the differentiation pathway, we would expect that the number of undifferentiated PGCs would decrease in *goe* mutants. However, the number of undifferentiated PGCs remained unchanged in *goe^5–11/331^* ovaries, resulting in a net increase in the total number of germ cells (Figure 4A). It is unlikely that this compensation for excess PGC differentiation in *goe* mutant ovaries was due to increased PGC proliferation or reduced cell death, because neither mitotic index of PGCs nor apoptotic profile showed any detectable changes in *goe^5–11/331^* ovaries (Figure 4H, I). Rather, because we observed an increase in the number of germline cysts undergoing dedifferentiation in *goe^5–11/331^* ovaries (Figure 4E–G), it is likely that compensation for excess PGC differentiation resulted from active dedifferentiation from differentiating germline cysts into PGCs. *goe^5–11/331^* mutant ovaries contained almost twice the normal number of dedifferentiating 2-cell cysts (Figure 4G). Because one developing germline cyst can dedifferentiate and break apart into multiple undifferentiated PGCs [25], dedifferentiation from a small number of differentiating germ cells could restore the PGC population, resulting in a net increase in total germ cell number. We conclude that the absence of Goe leads to excess PGC differentiation, but that the reduction in the PGC pool is partially compensated by dedifferentiation of 2-cell cysts. Taken together, our results suggest that the main function of Goe in the germline is to limit PGC differentiation in LL3 ovaries; an additional role in regulating dedifferentiation cannot be ruled out.

The ability of Goe to suppress PGC differentiation raises the possibility that Goe might regulate the timing of gametogenesis. However, we never detected precocious PGC differentiation in *goe^5–11/331^* ovaries before LL3 (Figure S5). In addition, we observed Goe expression both before and after the initiation of gametogenesis; Goe protein became detectable on the germ cell membrane by LL2, and continued to be expressed throughout the larval period (Figure S5). These observations suggest that Goe does not provide temporal information itself, but instead defines the fraction of PGCs that differentiate when the temporal signal provided by ecdysone initiates direct gametogenesis.

### *gone early* is expressed in EGF-producing cells in various tissues and can alleviate EGFR signaling activity

**Figure 5:**
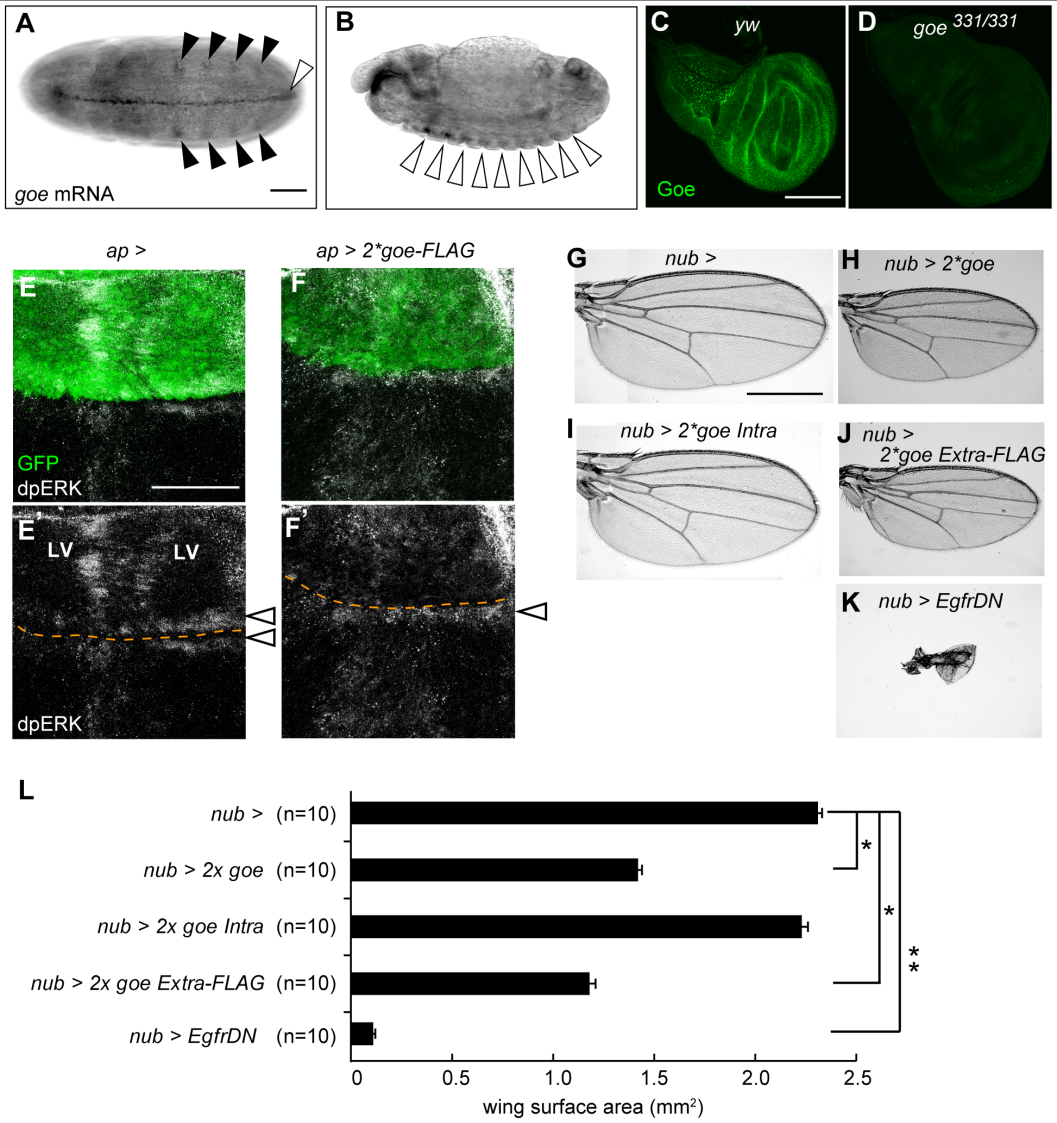
*gone early* is expressed in various EGF ligand-producing cells and can attenuate EGFR signaling. (A, B) Expression patterns of *goe* at embryonic stages. *goe* mRNA was expressed in ventral midline (white arrowhead in A), tracheal placodes (black arrowheads in A), and cells forming the anterior-most row in each segment (white arrowheads in B). Note that all of these regions are reported to produce and secrete EGF ligands [36]–[39]. Anterior is to the left. (A) Ventral view of an embryo at the stage of germ band elongation. (B) Lateral view of an embryo at the stage of germ band retraction. (C, D) *goe* expression in wing disc at LL3. Goe protein (green) was expressed ubiquitously in wing disc (C), but expression was abolished in the *goe^331/331^* mutant (D). Note that *spitz*, a gene encoding a major EGF ligand, exhibits a similar ubiquitous expression pattern [50]. (E–F′) LL3 wing imaginal discs stained for GFP and di-phosphorylated ERK (dpERK). The dorsal compartment of a wing disc was labeled with *UAS-GFP* expression driven by a dorsal compartment–specific gal4, *ap-gal4* [15] (green, E, F). (E, E′) A control *ap*> wing disc. Wing margin (open arrowheads, E) and vein primordia (LV, E′) were prominently labeled by dpERK (white, E′). (F, F′) A wing disc overexpressing *goe-FLAG* in the dorsal compartment (*ap>2x goe-FLAG*). dpERK staining in the wing margin remained detectable in the ventral compartment (white arrowhead, F′), but was ablated in the dorsal region (F′). dpERK staining was also reduced remarkably in vein primordia in the dorsal compartment (F′; compare with LV in E′). Such evident reduction of dpERK staining in the dorsal compartment was detected in 50% of the *ap>2x goe-FLAG* discs (n = 16), whereas it was never detected in controls (0%, n = 9, p>0.02, Fisher's exact probability test). Orange dotted lines represent boundaries between dorsal and ventral compartments. (G–K) Adult wings from control flies carrying a strong Gal4 wing driver, *nub-gal4* [15], alone (*nub*>) (G), flies expressing two copies of full-length *goe* cDNA (*nub>2x goe*) (H), *goe* without the extracellular region (*nub>2x goe Intra*) (I), or FLAG-tagged *goe* without the intracellular region (*nub>2x goe Extra-FLAG*) (J), and flies expressing the dominant negative form of *Egfr* (*nub>EgfrDN*) (K). (L) Average wing surface area in adult flies. Wing surface area was reduced in *nub>2x goe Extra-FLAG* flies to a level comparable to that of *nub>2x goe* flies (p = 0.00021, U-test), indicating that the extracellular region of Goe is necessary and sufficient to induce a small-wing phenotype. Wing surface area was measured using ImageJ; 10 wings were examined for each genotype (*p<0.0001, **p<0.00004; U-test). Scale bar: 50 µm (A, E), 100 µm (C), 500 µm (G).

To obtain further insights into the function of Goe, we surveyed the expression of *goe* in tissues other than the ovary. In embryos, *goe* was expressed in the ventral midline, tracheal placodes, and anterior-most row in each segment (Figure 5A, B). Strikingly, all of these regions produce EGF [36]–[39]. PGCs in the larval ovary, which express *goe* (Figure 1C–F), also secrete EGF [7], [40]. Given the opposing effects of Goe and EGFR signaling on PGC differentiation in LL3 ovaries (Figure 2, 4) [7], these observations raised the possibility that *goe* may somehow antagonize EGFR signaling activity.

To test this idea, we next focused on the wing imaginal disc, in which the role of EGFR signaling has been well characterized. At LL3, *goe* was expressed uniformly in the wing disc, whereas EGFR signaling was strongly activated in vein primordia, leading to prominent expression of di-phosphorylated ERK (dpERK), an indicator of EGFR signaling activation [41] (Figure 5C–E′). When *goe* was overexpressed in the dorsal compartment of the wing disc by *ap-gal4* (*ap>goe*), the prominent dpERK expression was compromised specifically in the dorsal compartment (Figure 5F, F′). This result shows that over-expression of *goe* can attenuate EGFR signaling activity. Consistent with this, overexpression of *goe* in entire wing discs caused a reduction in adult wing size, a weaker form of the phenotype caused by attenuating EGFR signaling [42] (Figure 5G, H, K, and L). A comparable small-wing phenotype was achieved when Goe fragment lacking the intracellular region was overexpressed, but such a phenotype was never observed when the extracellular region was deleted (Figure 5I, J, and L). These results show that Goe can negatively regulate EGFR signaling, and that its extracellular region mediates this ability.

The expression pattern of Goe may provide additional insight in the regulation of the EGFR signaling pathway by its negative regulators. Whereas all previously known negative regulators of the EGFR pathway (*argos*, *kekkon-1*, and *sprouty*) are expressed in signal-receiving cells and serve to fine-tune the spatial pattern of EGFR signal activation [43], Goe is unique in that it is expressed and required only in ligand-producing cells. The localization of Goe protein on the surface of cells contacting ligand-receiving IC cells raises the possibility that Goe may attenuate EGFR signaling by trapping EGF ligands at the surface of ligand-producing cells, thereby reducing the amount of EGF received by the receptor. Alternatively, Goe may interact with cell-intrinsic negative regulators acting at the surface of signal-receiving cells to facilitate their inhibitory effects [44]–[46].

### *gone early* and *argos* cooperatively suppress PGC differentiation to secure the minimal size of GSC precursor pool in LL3 ovaries

**Figure 6:**
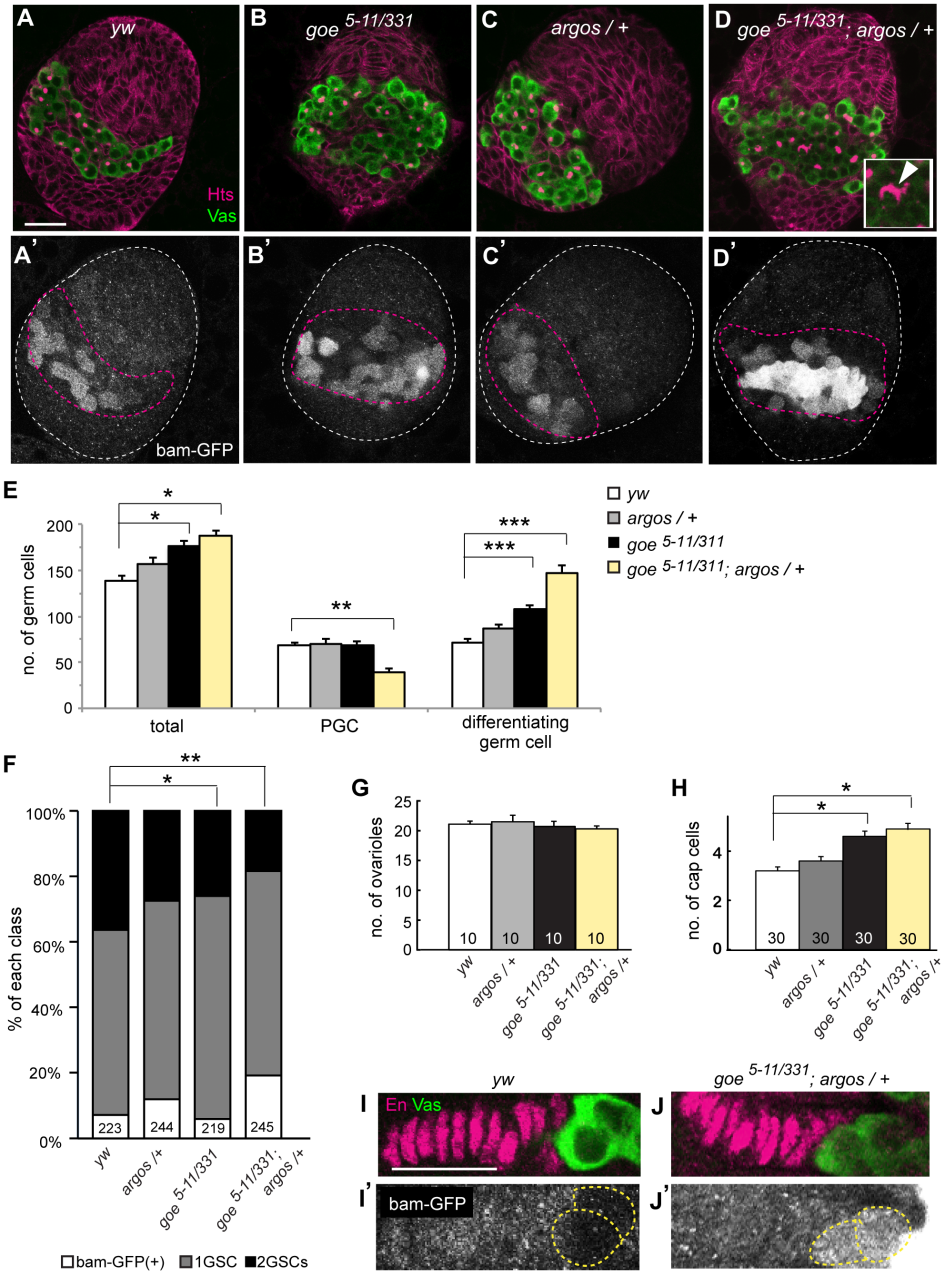
Genetic interaction between *gone early* and *argos* in the ovary. (A–D′) All confocal images depict LL3 ovaries triple-stained for Vasa (green, A–D), Hts (magenta, A–D), and GFP (*bam-GFP*) (white, A′–D′). (A, A′) A *y w* control ovary. (B, B′) A *goe^5–11/331^* ovary. (C, C′) An *argos* heterozygote ovary (*argos^delta7/+^*). (D, D′) A *goe^5–11/331^* ovary with one copy of *argos* (*goe^5–11/331^; argos^delta7/+^*). Inset in D shows an 8-cell cyst harboring a highly branched fusome (white arrowhead). White and magenta dashed lines in A′–D′ outline whole ovaries and GC/IC regions, respectively. (E) Average numbers of total germ cells, PGCs, and differentiating germ cells at LL3 in wild-type (white bars, *y w*), *argos^delta7/+^* (gray bars), *goe^5–11/331^* (black bars), and *goe^5–11/331^; argos^delta7/+^* (pale yellow bars) ovaries. P values were calculated vs. *y w* control by U-test (*p<0.001, **p<0.0005, ***p<5.0e-05). Between 9 and 23 ovaries were examined for each data point. (F) Distribution of the number of GSCs at WP in *y w, argos^delta7/+^*, *goe^5–11/331^*, and *goe^5–11/331^; argos^delta7/+^* ovarioles. P values were calculated using the chi-square test (*p = 0.004, **p = 1.0e-05). (G) Average numbers of ovarioles at WP in *y w, argos^delta7/+^, goe^5–11/331^*, and *goe^5–11/331^; argos^delta7/+^* ovaries. (H) Average numbers of cap cells at WP in *y w, argos^delta7/+^, goe^5–11/331^*, and *goe^5–11/331^; argos^delta7/+^* ovarioles. (*p<1.0e-05, U-test). The numbers of ovarioles (F, E, H) and ovaries (G) examined are indicated at the bottom of each bar. (I–J′) All confocal images depict WP ovarioles. Ovarioles were triple-stained for Vasa, En, and GFP. (I, J) Vasa labeled GSCs (green), and En labeled terminal filaments and cap cells (magenta). (I′, J′) Differentiation states of germ cells contacting cap cells (yellow dashed lines), considered to be GSCs or CB based on the absence or presence of *bam-GFP* expression (white), respectively. (I, I′) A *y w* control ovariole. (J, J′) A *goe^5–11/331^; argos^delta7/+^* ovariole. Although no GSCs in *y w* control ovarioles expressed *bam-GFP* (I′), all GSCs in *goe^5–11/331^, argos^delta7/+^* ovarioles expressed *bam-GFP* (J′). Anterior in A–D′ and I–J′ is up and to the left, respectively. Error bars indicate SEM. Scale bar: 20 µm.

Based on the potential regulatory function of Goe on EGFR signaling, we examined the genetic interactions between *goe* and other regulators of EGFR signaling in LL3 ovaries. ICs in these ovaries expressed Argos, an antagonist of EGF (Figure S6) that is secreted from EGFR signal–receiving cells into the extracellular matrix and sequester EGF ligands, thereby attenuating EGFR signaling [43]–[45]. We investigated whether *goe* and *argos* genetically interact to suppress PGC differentiation. Halving the gene dose of *argos* in a *goe* mutant background (*goe^5–11/331^; argos^delta7/+^*) synergistically increased the number of differentiating germ cells (Figure 6A–E). Although the shift toward differentiation in the *goe* single mutant was corrected through dedifferentiation, this compensatory pathway was apparently not adequate to cope with the massive differentiation caused by decreasing the *argos* gene dosage, and therefore led to a reduction in the PGC pool size (Figure 6E). These observations demonstrate that Goe and Argos, which are expressed in the germline and soma, respectively, cooperatively prevent excessive PGC differentiation and thereby secure the minimal size of the PGC pool.

We next examined the impact of reducing the PGC pool size on GSC establishment in the *goe^5–11/331^; argos^delta7/+^* ovary. Although neither *goe^5–11/331^* nor *argos^delta7/+^* ovaries exhibited a remarkable reduction in the number of GSCs at WP, GSC establishment was severely impaired in the *goe^5–11/331^; argos^delta7/+^* ovary (Figure 6F, I–J′). This reduction in GSC number likely resulted from an insufficient PGC pool, because both ovariole formation and niche development occurred normally in the *goe^5–11/331^; argos^delta7/+^* ovary (Figure 6G, H). Taken together, our results indicate that the cooperative function of *goe* in the germline and *argos* in somatic stromal cells is essential for restricting PGC differentiation at LL3, thereby securing the absolute number of ovarian GSCs.

## Conclusions

In *Drosophila* ovary and testis and mice testis, PGCs contribute to gamete production through two pathways: by remaining undifferentiated to give rise to GSCs, or by differentiating directly to gametes [1], [2]. Many previous studies have shown that such PGC fate decisions are primarily dependent on somatic stromal environment [2], [7], [9], [47]. Here, we propose that germ cells also contribute to the decision-making mechanism. We present genetic evidence that Goe on germ cell plasma membranes, acting cooperatively with an antagonist of EGFR signaling on stromal ICs, limits PGC differentiation and thereby secures the absolute number of GSCs. Goe could potentially act cell-autonomously to regulate PGC differentiation in a dose dependent fashion. However, we did not find indications that Goe expression levels differ between PGCs and differentiating germ cells; instead, Goe was expressed at comparable levels in all germ cells (Figure 1C–F). We thus hypothesize that rather than having a direct effect on PGC decision-making, Goe accelerates down-regulation of EGFR signaling on ICs in the entire GC/IC region, and the resultant low level of EGFR signaling limits the proportion of PGCs starting gametogenesis; the low level of EGFR signaling maintained by Goe increases the frequency of Dally-expressing ICs throughout the entire GC/IC region, and this in turn enhances the posterior diffusion of Dpp and expands the undifferentiated PGC pool posteriorly [7]. This idea is supported by the potential role of Goe as an antagonist of EGFR signaling and the synergistic interaction between *goe* and *argos* in suppressing PGC differentiation.

Goe was not uniformly distributed on the germ cell plasma membrane, but was instead localized to a domain of the membrane at the interface between germ cells. This is the first demonstration that the membrane domain at the interface between germ cells has a different molecular context than other regions of the germ cell membrane. Because *goe* is required to limit PGC differentiation, this observation suggests that the Goe-bounded membrane domain at the interface between germ cells is the site of cell–cell communication that is essential for prevention of PGC differentiation. Such communication may take place between neighboring germ cells. However, given that IC processes frequently penetrate into germ cell interfaces, we favor the possibility that Goe mediates communication between germ cell–IC processes at interfaces between germ cells.

Goe is essential for preventing a shift of the germ cell population to direct gametogenesis, but is dispensable for reconstitution of the PGC pool by dedifferentiation. We propose that Goe suppresses the allocation of PGCs to direct gametogenesis, thereby securing the fraction of ‘naive GSCs’ that have never experienced dedifferentiation during development. In the *Drosophila* testis, GSCs that have experienced dedifferentiation can function as stem cells, but exhibit a high frequency of centrosome misorientation and divide less frequently. The accumulation of such GSCs is one cause of the age-related decline in gamete production [48]. Therefore, Goe-mediated suppression of the direct gametogenesis pathway represents a strategy for maintaining the integrity of the GSC system, both in number and quality.

## Supporting Information

Figure S1 Thin cell processes of ICs intervene between germ cells. (A, B) TEMs showing the anterior end of GC/IC region in an LL3 ovary. In B, ICs and terminal filament cells are pseudocolored in pink and yellow, respectively. ICs extend a long thin cell process between germ cells in the GC/IC region. Red asterisk indicates a cytoplasmic bridge in a germ cell undergoing cytokinesis. Scale bar: 2 µm.
(TIF)

Figure S2 Behavior of germ cells in *nos>goe* ovaries. (A–B″) All confocal images depict LL3 ovaries triple-stained for GFP (*bam-GFP*), Hts, and Vasa. (A, A′, A″) An ovary identical to that shown in Fig. 2C and C′. (B, B′, B″) An ovary identical to that shown in Fig. 2D and D′. (A, B) Merged images of Vasa (magenta) and GFP (green). Note that the number of *bam-GFP*-negative germ cells (PGCs) increased in *nos>goe* ovaries (B) relative to that in *nos*> control ovaries (A), whereas the number of *bam-GFP*-positive germ cells (differentiating germ cells) decreased. (A′, B′) Merged images of Hts (magenta) and GFP (green). Note that *bam-GFP*-negative germ cells contained spherical or dumbbell-shaped fusomes but never U-shaped or branched fusomes, suggesting that these cells were single or dividing PGCs. (A″, B″) Images shown in Fig. 2C and 2D; Hts (magenta), Vasa (green). Scale bar: 20 µm.
(TIF)

Figure S3 Behavior of germ cells in *goe* mutant ovaries. (A–C″) All confocal images depict LL3 ovaries triple-stained for GFP (*bam-GFP*), Hts, and Vasa. (A, A′, A″) An ovary identical to that shown in Fig. 4B and B′. (B, B′, B″) An ovary identical to that shown in Fig. 4C and C′. (C, C′, C″) An ovary identical to that shown in Fig. 4D and D′. (A, B, C) Merged images of Vasa (magenta) and GFP (green). (A′, B′, C′) Merged images of Hts (magenta) and GFP (green). (A″, B″, C″) Images shown in Fig. 4B, C, and D; Hts (magenta), Vasa (green). Note that highly differentiated germ cell cysts (white arrowheads: 4-cell cysts, red arrowheads: 8-cell cysts) were observed in *goe* mutant ovaries (B–B″) but not in *y w* control (A–A″) or rescued ovaries (C–C″), supporting the idea that Goe is required to suppress PGC differentiation. The highly differentiated cysts could be distinguished from CB and 2-cell cysts by their stronger expression of *bam-GFP* and U-shaped or branched fusomes. Scale bar: 20 µm.
(TIF)

Figure S4 Dot-like fusomes between germ cells are localized in ring canal remnants. (A) Co-localization of dot-like fusome (Hts, magenta, orange arrowhead) with Pav-GFP, a component of a ring canal remnant (GFP, green, white arrowhead) in an LL3 ovary. Scale bar: 10 µm. (B) The average number of cells with a dot-like fusome between two connecting cells (light gray and black bars) was almost identical to the number with a Pav-GFP–marked ring canal remnant (white and gray bars), demonstrating that a dot-like fusome is a reliable marker for cells with ring canal remnants. The number of ovaries examined is indicated at the bottom of each bar. Error bars indicate SEM.
(TIF)

Figure S5 Premature PGC differentiation never occurs in *goe* mutant ovaries. (A) Distribution of PGC, CB, and cyst (2- to 16-cell cysts) in *goe^5–11/331^* ovaries at LL2. No differentiating germ cells were observed in *y w* control or *goe^5–11/331^* ovaries. The numbers of germ cells and ovaries examined are indicated at the bottom of each bar and in parentheses, respectively. (B–C′) Ovaries were triple-stained for Vasa, Hts, and GFP (*bam-GFP*). (B, C) Vasa labeled germ cells (green), and Hts outlined somatic cells and fusomes (magenta). (B′, C′) Differentiating germ cells were marked by *bam-GFP*. (B, B′) A *y w* control ovary. (C, C′) A *goe^5–11/331^* ovary. White and magenta dashed lines in B′ and C′ outline whole ovaries and GC/IC regions, respectively. (D) A *y w* ovary stained for Vasa (magenta) and Goe (green). Anterior is up. Scale bar: 20 µm.
(TIF)

Figure S6 *argos* is expressed in ICs in LL3 ovaries. (A) An ovary stained for Vasa (magenta) and *argos* mRNA (green). *argos* mRNA was detected in ICs, but not in germ cells. (B) No signal was observed in a sense probe control. Insets show magnified views of GC/IC regions. White dashed lines outline whole ovaries. Anterior is up. Scale bar: 20 µm.
(TIF)
