# Supplementary figures and images for: gone early, a Novel Germline Factor, Ensures the Proper Size of the Stem Cell Precursor Pool in the Drosophila Ovary
Source: PLoS One. 2014 Nov 24;9(11):e113423. doi: 10.1371/journal.pone.0113423 (PMC4242634; doi:10.1371/journal.pone.0113423)

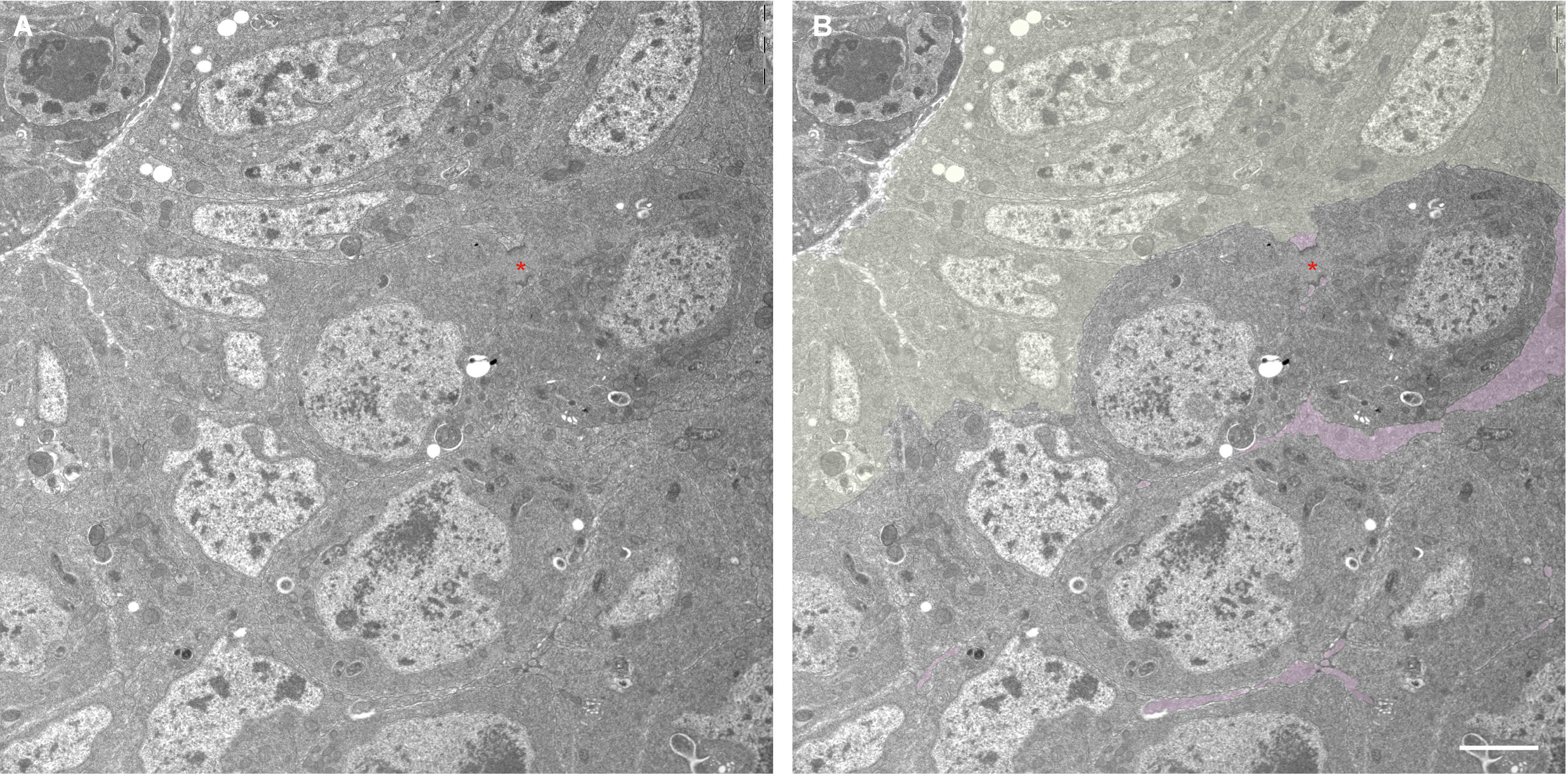

Supplement: Figure S1 — Thin cell processes of ICs intervene between germ cells. (A, B) TEMs showing the anterior end of GC/IC region in an LL3 ovary. In B, ICs and terminal filament cells are pseudocolored in pink and yellow, respectively. ICs extend a long thin cell process between germ cells in the GC/IC region. Red asterisk indicates a cytoplasmic bridge in a germ cell undergoing cytokinesis. Scale bar: 2 µm. (TIF) [file pone.0113423.s001.tif]

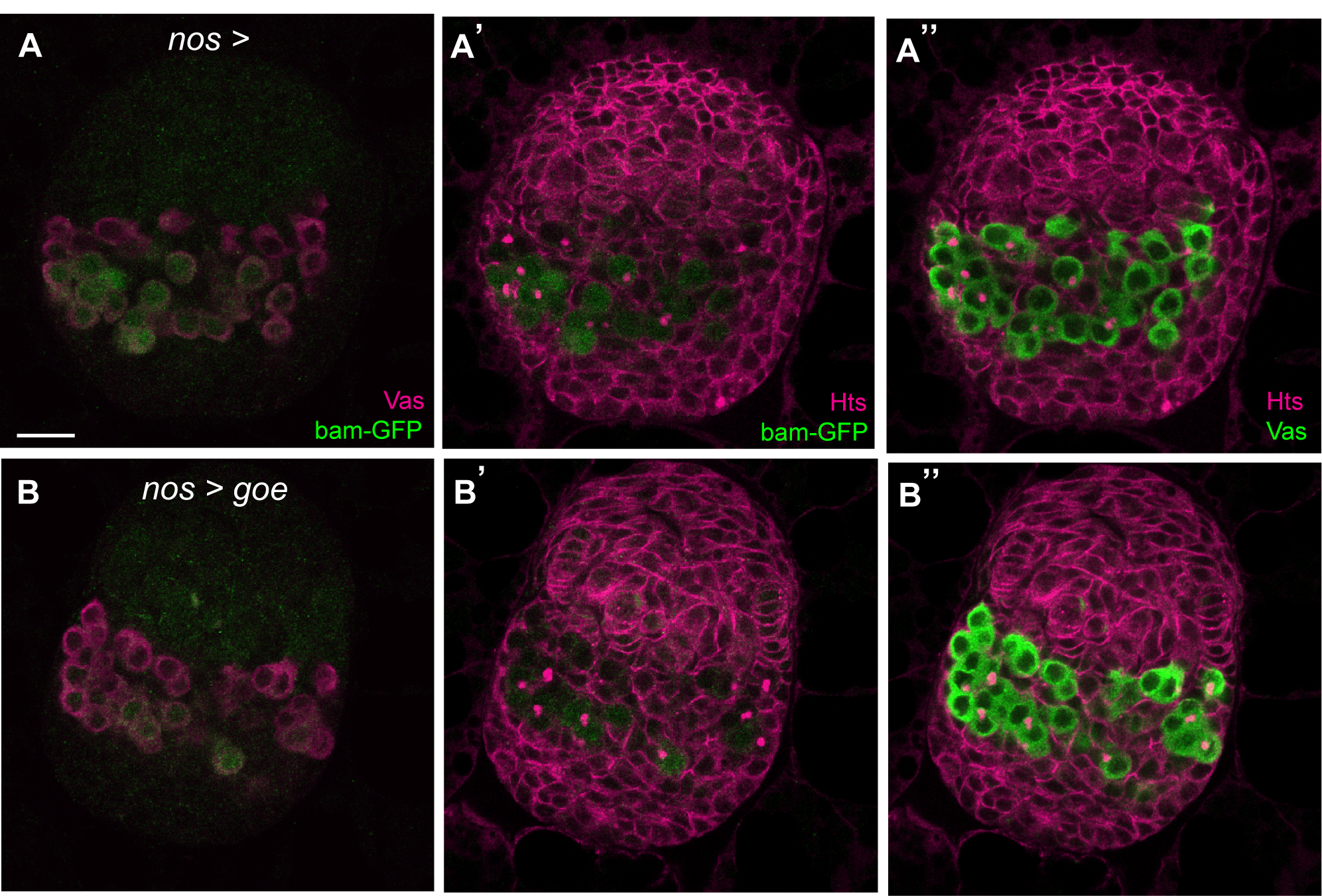

Supplement: Figure S2 — Behavior of germ cells in nos>goe ovaries. (A–B″) All confocal images depict LL3 ovaries triple-stained for GFP (bam-GFP), Hts, and Vasa. (A, A′, A″) An ovary identical to that shown in Fig. 2C and C′. (B, B′, B″) An ovary identical to that shown in Fig. 2D and D′. (A, B) Merged images of Vasa (magenta) and GFP (green). Note that the number of bam-GFP-negative germ cells (PGCs) increased in nos>goe ovaries (B) relative to that in nos> control ovaries (A), whereas the number of bam-GFP-positive germ cells (differentiating germ cells) decreased. (A′, B′) Merged images of Hts (magenta) and GFP (green). Note that bam-GFP-negative germ cells contained spherical or dumbbell-shaped fusomes but never U-shaped or branched fusomes, suggesting that these cells were single or dividing PGCs. (A″, B″) Images shown in Fig. 2C and 2D; Hts (magenta), Vasa (green). Scale bar: 20 µm. (TIF) [file pone.0113423.s002.tif]

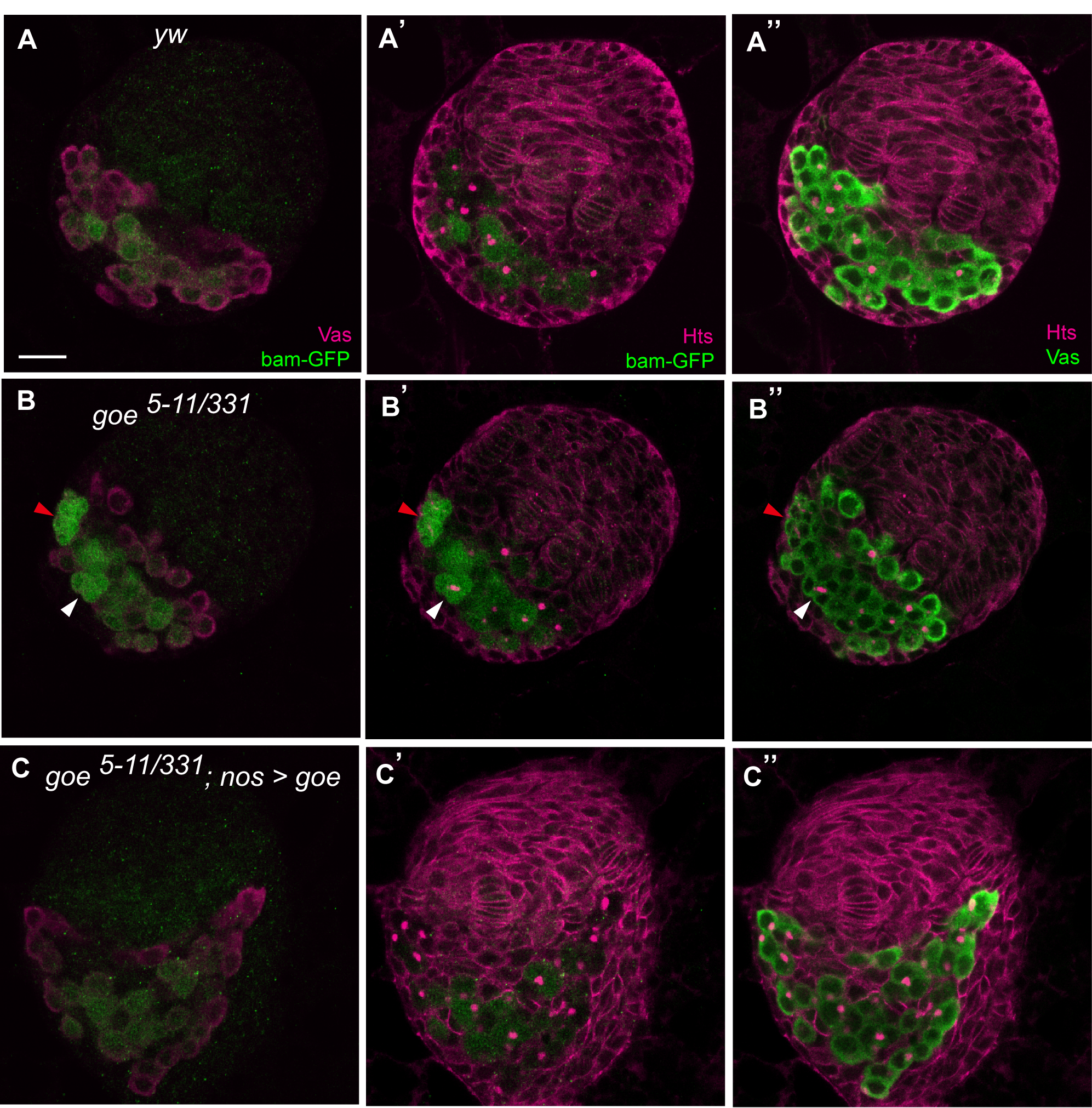

Supplement: Figure S3 — Behavior of germ cells in goe mutant ovaries. (A–C″) All confocal images depict LL3 ovaries triple-stained for GFP (bam-GFP), Hts, and Vasa. (A, A′, A″) An ovary identical to that shown in Fig. 4B and B′. (B, B′, B″) An ovary identical to that shown in Fig. 4C and C′. (C, C′, C″) An ovary identical to that shown in Fig. 4D and D′. (A, B, C) Merged images of Vasa (magenta) and GFP (green). (A′, B′, C′) Merged images of Hts (magenta) and GFP (green). (A″, B″, C″) Images shown in Fig. 4B, C, and D; Hts (magenta), Vasa (green). Note that highly differentiated germ cell cysts (white arrowheads: 4-cell cysts, red arrowheads: 8-cell cysts) were observed in goe mutant ovaries (B–B″) but not in y w control (A–A″) or rescued ovaries (C–C″), supporting the idea that Goe is required to suppress PGC differentiation. The highly differentiated cysts could be distinguished from CB and 2-cell cysts by their stronger expression of bam-GFP and U-shaped or branched fusomes. Scale bar: 20 µm. (TIF) [file pone.0113423.s003.tif]

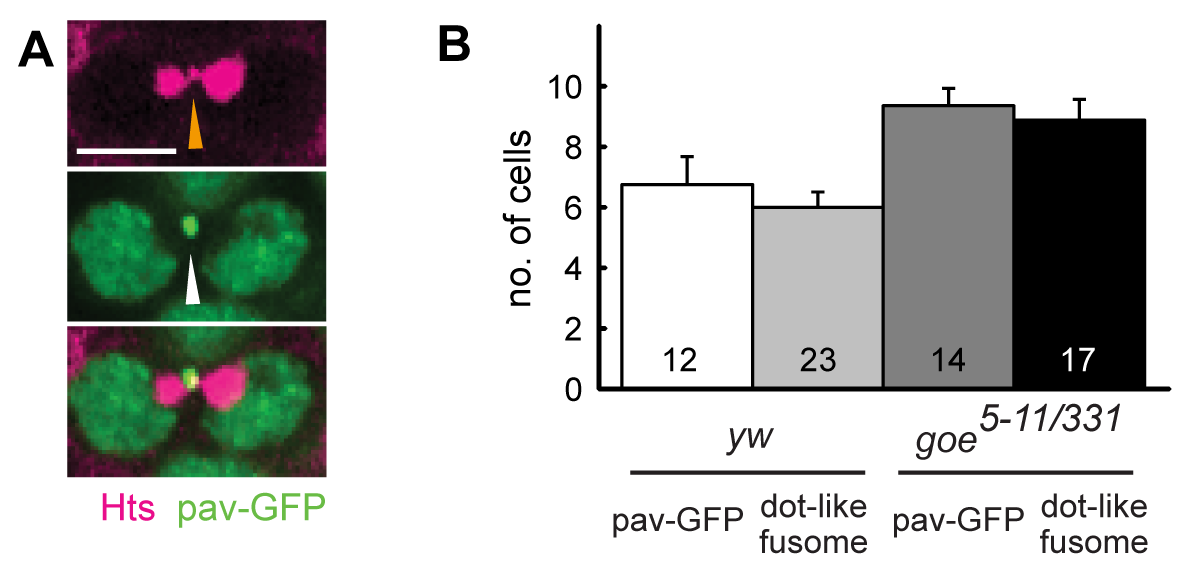

Supplement: Figure S4 — Dot-like fusomes between germ cells are localized in ring canal remnants. (A) Co-localization of dot-like fusome (Hts, magenta, orange arrowhead) with Pav-GFP, a component of a ring canal remnant (GFP, green, white arrowhead) in an LL3 ovary. Scale bar: 10 µm. (B) The average number of cells with a dot-like fusome between two connecting cells (light gray and black bars) was almost identical to the number with a Pav-GFP–marked ring canal remnant (white and gray bars), demonstrating that a dot-like fusome is a reliable marker for cells with ring canal remnants. The number of ovaries examined is indicated at the bottom of each bar. Error bars indicate SEM. (TIF) [file pone.0113423.s004.tif]

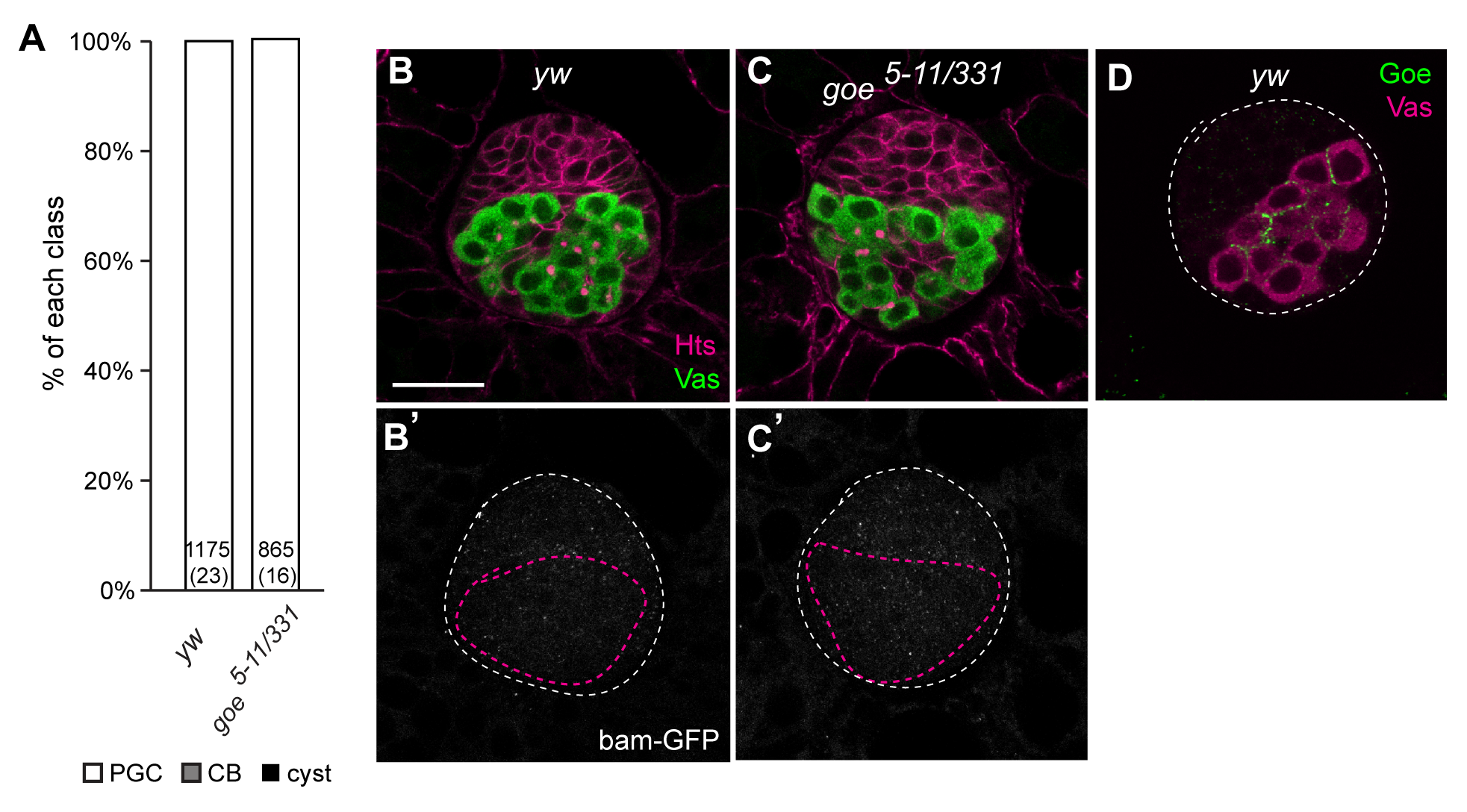

Supplement: Figure S5 — Premature PGC differentiation never occurs in goe mutant ovaries. (A) Distribution of PGC, CB, and cyst (2- to 16-cell cysts) in goe5–11/331 ovaries at LL2. No differentiating germ cells were observed in y w control or goe5–11/331 ovaries. The numbers of germ cells and ovaries examined are indicated at the bottom of each bar and in parentheses, respectively. (B–C′) Ovaries were triple-stained for Vasa, Hts, and GFP (bam-GFP). (B, C) Vasa labeled germ cells (green), and Hts outlined somatic cells and fusomes (magenta). (B′, C′) Differentiating germ cells were marked by bam-GFP. (B, B′) A y w control ovary. (C, C′) A goe5–11/331 ovary. White and magenta dashed lines in B′ and C′ outline whole ovaries and GC/IC regions, respectively. (D) A y w ovary stained for Vasa (magenta) and Goe (green). Anterior is up. Scale bar: 20 µm. (TIF) [file pone.0113423.s005.tif]

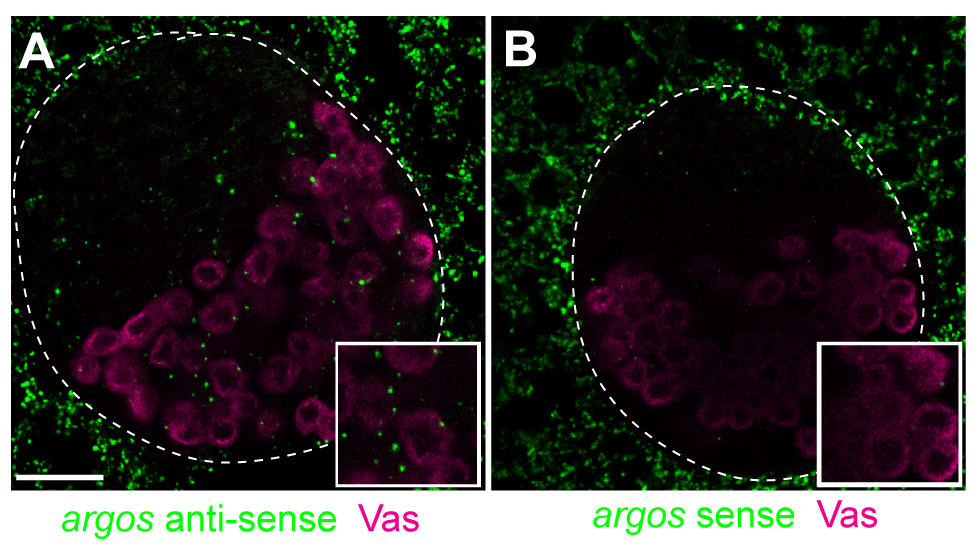

Supplement: Figure S6 — argos is expressed in ICs in LL3 ovaries. (A) An ovary stained for Vasa (magenta) and argos mRNA (green). argos mRNA was detected in ICs, but not in germ cells. (B) No signal was observed in a sense probe control. Insets show magnified views of GC/IC regions. White dashed lines outline whole ovaries. Anterior is up. Scale bar: 20 µm. (TIF) [file pone.0113423.s006.tif]
